# Can Positive Emotions Predict Consumer Satisfaction in Adverse Services?

**DOI:** 10.12688/f1000research.110256.1

**Published:** 2022-03-22

**Authors:** Nahima Akthar, Dr. Smitha Nayak, Dr. Yogesh Pai P

**Affiliations:** 1 Manipal Institute of Management, Manipal Academy of Higher Education, Udupi, Karnataka, 576104, India

**Keywords:** Happiness, mood, patient satisfaction, perceived service quality, negative services

## Abstract

**Background:** Managing emotions during hospital visits is important to enhance patient satisfaction. The purpose of this paper is to explore the relationship between patients’ happiness and satisfaction through patients’ mood and perceived service quality at a healthcare setup.

**Methods:** This study was conducted in a tertiary care hospital located in coastal Karnataka during the period from November to December 2021. Primary data was collected through a structured questionnaire from 227 respondents. “Statistical Package for the Social Sciences (SPSS) 27.0” and “SmartPLS 3.0” software was used for data analysis.

**Results:** Hypotheses proposed in this study were examined by comparing the direct effect of patients’ happiness level on perceived service quality and the indirect effect of the level of patients’ happiness on patient satisfaction. The influence of all the exogenous latent variables namely, happiness, mood, perceived service quality, on the endogenous latent variable of patient satisfaction is estimated to be moderate (R ^2^=62.5%)

**Conclusion:** This study empowers hospital managers to recognize how patient satisfaction is dependent on patients’ happiness. In order to enhance patient satisfaction, the quality of care provided by health services, human resources, and infrastructure must be improved. As a result, the entire service encounter can be made enjoyable to the customers by reducing the distress caused by adverse services. Managers can utilize the outcomes of the study to develop marketing strategies to influence patients’ emotions in the healthcare setup by modifying the servicescape elements.

## Introduction

The service sectors are growing faster as compared to other sectors in developing nations ( Mukherjee, 2015; Latha, 2016; Service sectors in India, 2021). The services industry not only accounts for the bulk of India’s Gross Domestic Product (GDP), but it also attracts significant foreign investment, contributes significantly to export, and employs a vast majority of the population. In the financial year 2021, the services sector in India contributed 54 percent of the total Gross Value Added. Hence the service sector is an integral component of economic growth in India and necessitates investigation on various economic and behavioral dimensions.

The service industry comprises routine, positive, and negative services. “Routine or neutral services” raise familiar experiences that they navigate frequently. For example, the housekeeping and beauty services. “Positive services” are those associated with travel and entertainment. Tourism and hospitality services are examples of positive services. “Negative services” are those associated with unpleasant circumstances. Law and healthcare services are examples of negative services ( Morgan & Rao, 2006).

The healthcare industry is one of the fastest-growing service industries in recent years. In the last few decades, extensive attempts have been made to incorporate “information and communication technology” (ICT) into healthcare operations ( Fullman *et al.*, 2018; Aayog, 2019). It is projected that India’s healthcare industry is expected to grow up to 372 billion dollars by 2022. The country’s healthcare market had grown rapidly to become one of the largest sectors in terms of income and jobs ( Statista Research Department, 2021). This necessitates studies on the healthcare sector.

The services offered by law and healthcare are considered to be unpleasant ( Hellén & Sääksjärvi, 2011). These services are referred to as “adverse services” or “negative services” since the clients are exposed to uncomfortable situations such as overcrowding, long waiting times, anxiety, and risk of infections. In adverse services customers are exposed to these situations which cause an element of uneasiness which further influences their service experience ( Morgan & Rao, 2006; Schwartz, 2015; Bahadori, Teymourzadeh, Ravangard, & Raadabadi, 2017). Although healthcare services are hostile, many people are forced to use them at some point in their lives ( Hellén & Sääksjärvi, 2011). Negative emotional states such as worry, uncertainty, and unease are frequently created in hospital settings ( Morgan & Rao, 2006; Berry & Bendapudi, 2007; Miller, Luce, Kahn, & Conant, 2009). Researchers have examined the association between emotions and service quality evaluations ( Mattila & Enz, 2002; Slåtten, 2011; Naami, & Hezarkhani, 2018). Ben-Ze’ev (2000) opined that positive emotions such as joy or happiness express satisfaction, while negative emotions such as rage or guilt express dissatisfaction.

Scholars in the field of “positive psychology” have defined happiness as “a summary appraisal of one’s life” ( Diener, Scollon, & Lucas, 2009). Happiness is described as a feeling, sentiment, or transitory form of joy ( Labroo & Patrick, 2008; Labroo & Mukhopadhyay, 2009). An individual’s happiness level impacts how happy and unhappy people acquire, understand, and evaluate the same situation. The happiness level of the consumer predicts their ability to cope in difficult conditions ( Boehm, Ruberton, & Lyubomirsky, 2017). According to past research, consumers’ attitudes vary depending on the services they have received (positive or negative). Happiness, on the other hand, seems to remain consistent over time and in various situations ( Hellén & Sääksjärvi, 2011). During service encounters, consumers have a strong tendency to pursue psychological needs such as hedonistic needs, inner joy, and happiness ( Boehm *et al*., 2017). Researchers revealed that consumers with positive emotional states are more likely to evaluate services in a positive way ( Ali, Amin, & Cobanoglu, 2016).

Service quality (SQ) evaluation is a cognitive process in which clients compare service quality expectations to the actual services obtained ( Lee, Lee, & Yoo, 2000; Abedniya, & Zaeim, 2011). Therefore, hospitals must ensure that good quality services are provided to meet their client’s expectations ( Suki, Lian, & Suki, 2011; Rechel, Doyle, Grundy, & McKee, 2009). Marketers need to explore the precursors of service quality evaluation. In service marketing literature, precursors of service quality evaluation by the customers have received considerable attention over the recent decade ( Kiran & Diljit, 2017; Tan, Benbasat, & Cenfetelli, 2013; Sultan & Wong, 2011; Shamdasani, Mukherjee, & Malhotra, 2008).

Service quality is critical for healthcare organizations, and it has a significant impact on patient satisfaction ( Dagger, Sweeney, & Johnson, 2007; Zaim, Bayyurt, & Zaim, 2010). The major outcomes of a business are SQ and customer satisfaction (CS), in this case, patient satisfaction. Patients are treated as guests who are seeking positive outcomes as well as quality service experiences ( Otani, Waterman, Faulkner, Boslaugh, & Dunagan, 2010; Marzo *et al.*, 2021). Delivering exceptional services leads to a high CS, which leads to customer retention ( Otani *et al.*, 2010; Loureiro, Miranda, & Breazeale, 2014; Oluwafemi & Dastane, 2016).

Patient satisfaction (PS) is an individual opinion of the standard of care received ( Otani, Kurz, Harris, & Byrne, 2005). PS is a critical analysis of patients’ happiness with the quality of health care they receive both in and out of the doctor’s office. PS is a major determinant of the quality of healthcare outcomes ( Marzo *et al.*, 2021). CS is essential to every industry since satisfied customers are loyal and bring in new business. The healthcare industry is no exception to this. Many studies believe that happy patients are more likely to tell their friends about their doctors and return when they need help again ( Otani *et al.*, 2010). CS reflects the feelings of healthcare patients about the quality of service they expect in comparison to what they currently experience. It’s also possible to presume that the satisfaction level of the patient is determined by the number of expectations and realities learned from the health services he/she has received ( Kotler & Keller, 2016).

In the field of psychology, the importance of emotions has acquired a lot of attention, but it is lacking in the marketing literature ( Hellén, 2010). Positive services are well researched when compared to negative services ( Morgan & Rao, 2006; Berry & Bendapudi, 2007; Miller *et al.*, 2009). Thus, the goal of this study is to determine the relationship between patients’ happiness and satisfaction through patients’ mood and perceived service quality at a healthcare setup. This research contributes to the service marketing literature by demonstrating how patient satisfaction is enhanced in an unpleasant service context by mood and perceived service quality. Healthcare consumers are susceptible to deal with the adverse features of the service differently and to evaluate the quality of service encounters through emotions to develop satisfaction. The hospital management can support healthcare consumers to improve satisfaction by changing the servicescape of the hospital.

This research article begins with a review of literature on the constructs of the study. In the subsequent sections the methodology adopted and the data analysis is presented followed by the major findings, limitations of the study, and directions for future research.

## Literature review

### Theoretical background

Ashby and Isen (1999) proposed that positive affect has a consistent impact on a variety of cognitive activities. Many of these effects are explained by the neuropsychological theory, which claims that the pleasant effect is linked to higher levels of dopamine in the brain. They have suggested that “positive affect influences olfaction, the consolidation of long-term or episodic memories, working memory, and creative problem-solving”. For example, the idea claims that higher dopamine release in the “anterior cingulate” promotes “cognitive flexibility” and allows the choice of “cognitive perspectives”, which helps with creative problem-solving. The resulting theory has several advantages over other methods to the study of positive affect now in use. First and foremost, it offers a neuropsychological explanation for a variety of well-known positive affect occurrences. Second, it predicts “positive affect” influences on tasks that have never been studied by the researchers. Third, it lists several tasks in which positive emotion is not expected to have an impact on performance. Fourth, it connects positive emotion research findings to previously unconnected neuropsychological studies. For example, it compares cognitive processing in healthy people with cognitive processing in some neuropsychological patient groups.

### Happiness

Subjective wellbeing (SWB) is a synonym for happiness ( Lu, Gilmour, & Kao, 2001; Chang & Nayga, 2010). In the positive psychology literature, happiness is defined as “a summary appraisal of one’s life” ( Diener *et al.*, 2009; Veenhoven, 2010). Diener stated that “the term happy in common English usage refers to a transient, positive state of mind brought on by a specific experience, such as a nice social engagement” ( Diener *et al.*, 2009). One of the most important aspects of a human being’s life is their mental state of mind ( Diener, Lucas, & Oishi, 2002).

Various factors influence happiness levels, such as individual feelings of joy, positive well-being, and a sense of a good and meaningful life ( Lyubomirsky & Ross, 1997; Lyubomirsky & Tucker, 1998; Lyubomirsky, Tucker, & Kasri, 2001). Lyubomirsky *et al*. (2001) and Lyubomirsky, King, and Diener (2005) have found that happy people make more positive decisions than unhappy people. As a result, happiness plays a crucial role in determining outcomes. Happy customers are more likely to be pleased when they make a specific decision. Happy people have a more consistent reaction to life situations, as they are considerably better at dealing with stressful conditions than unhappy people ( Hellén & Sääksjärvi, 2011). Happy people are more likely to be connected by more positive life circumstances, and as a result, they have more positive outcomes in their lives ( Kahneman, Krueger, Schkade, Schwarz, & Stone, 2006).

### Mood

Customers’ moods are transient states of emotion that prompt them to evaluate services precisely ( Pieters & Raaij, 1988). When compared to other emotional states that endure longer, mood has a lower intensity, is more distributed, and is unintentional ( Bagozzi *et al*., 1999). Lane and Terry (2000) have defined mood as “a collection of experiences that are fleeting in character, vary in intensity and length, and frequently involve multiple emotions”. This definition states that mood is a chain of expressive emotions that forms a frame of mind to change incoming events gradually through day-to-day activities, as happy customers are in a better mood, they have a greater opinion of service quality than disgruntled customers ( Hellén & Sääksjärvi, 2011). Therefore, happiness is defined as an emotional state of well-being that is stored in the memory as a mood rather than an emotion ( Belanche, Casaló, & Guinalu, 2013).

### Perceived service quality (PSQ)

“The customer’s assessment of an entity’s total excellence or superiority can be defined as service quality” ( Zeithaml, 1988). Consumers evaluate the service quality of an organization by comparing their perceptions to their expectations ( Sivakumar, Li, & Dong, 2014). Service providers must ensure that service recipients have pleasant service interactions, as negative experiences will be shared with others ( Petzer, Meyer, Svari, & Svensson, 2012). According to recent research in the service industry, negative emotions experienced by service recipients during the service contact have an impact on their loyalty levels ( Kiran & Diljit, 2017; Ali *et al.*, 2016; Park, Lee, Kwon, & Del Pobil, 2015; Son, Jung, & Lee, 2015).

Service quality is a critical factor in determining whether or not a service provider is favored, hence it must be carefully measured and improved ( Javed & Ilyas, 2018; Marzo *et al.*, 2021). According to researchers, hospitals must now meet their criteria and deliver the greatest health care services to patients as a result of growing expectations for common facilities ( Padma, Rajendran, & Lokachari, 2010; Pai & Chary, 2014). PSQ in the healthcare sector has received a lot of attention. It should be mentioned that in both public and private institutions, patients’ perceptions of healthcare services are influenced by the quality of care they receive ( Shabbir, Malik, Malik, & Wiele, 2016).

### Patient satisfaction (PS)

One of the most widely researched topics in literature is satisfaction ( Sawyer *et al.*, 2013; Barnett *et al.*, 2013). According to Oliver (2000), satisfaction is “a post-consumption judgment by the consumer that a service provides a pleasant level of consumption-related fulfillment, including under or over-fulfillment.” Patient satisfaction is one of the most often reported outcome indicators for quality of care in the healthcare sector, and it can be referred to as consumer satisfaction ( Marzo *et al.*, 2021). Patient satisfaction is described as “meeting or exceeding the requests and expectations of the patient” ( Akbulut, 2016; Yilmaz, 2011). This situation may arise as a result of patients’ inability to assess the medical element of the treatments provided. During the examination, physicians’ compassion, empathy, and other related abilities have a beneficial impact on patient satisfaction ( Akbolat, Sezer, Ünal, & Amarat, 2021). Patients who are happy with the service they receive will share their experience among people they know ( Juhana, Manik, Febrinella, & Sidharta, 2015).

**Figure 1.**
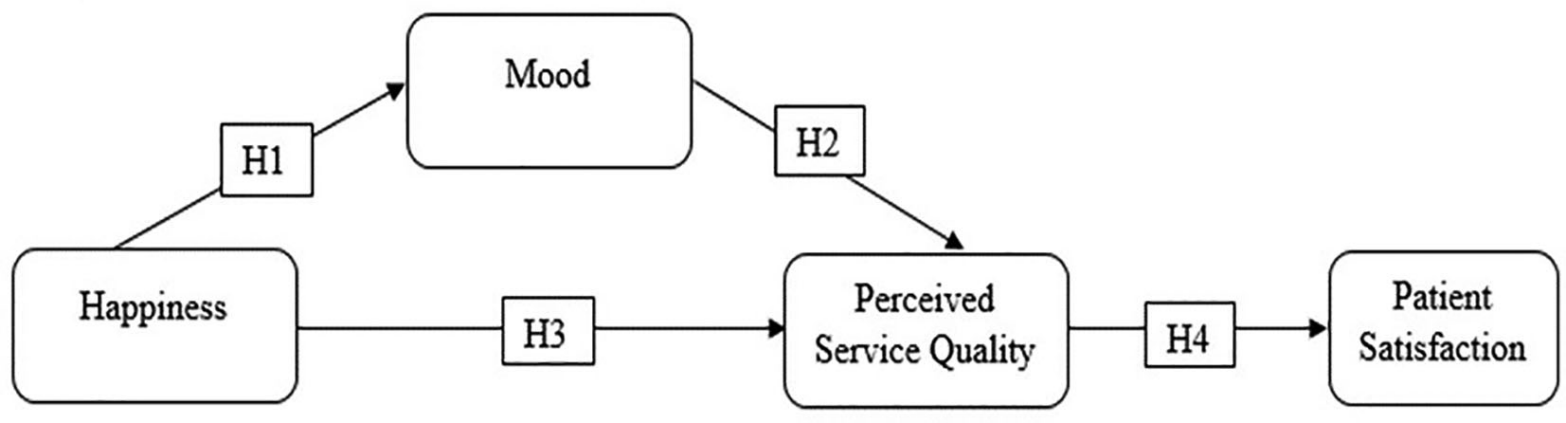
Proposed hypotheses.

The hypotheses proposed in this study are shown in Figure 1. The model proposes that patients’ happiness influences their satisfaction via mood and PSQ.

Happiness causes recurrent pleasant moods because happy persons are more likely to have optimistic ideas ( Lyubomirsky *et al*., 2005; Diener & Biswas-Diener, 2005). According to psychologists, a cheerful individual might experience a bad mood. Moods fluctuate to some extent as a result of positive and negative events. Even when their moods vary, the happy person adapts to events while maintaining a positive attitude ( Diener *et al.*, 2009). Happiness is important in healthcare services because it protects consumers from the harmful consequences of those services. Individuals who are happy cope with stress better and can maintain a positive attitude in adverse services ( Hellén & Sääksjärvi, 2011). As a result, the association between happiness and mood is likely to be substantial in healthcare services.

*H1*: *Happiness is positively related to mood in adverse services.*

Previous researchers have discovered an association between mood and PSQ ( Kocabulut & Albayrak, 2019; Pornpitakpan, Yuan, & Han, 2017; White, 2006). Experts have documented that the impact of mood is greatest when the buyer is uninformed of the goods or services. Individuals are more likely to rely on their feelings in uncertain situations ( Dubé, & Morgan, 1996). According to a comprehensive understanding, patients find it difficult to measure service quality in unpleasant services, thus they lean on their moods for assessment ( Collier, 1994). Similarly, Darke, Chattopadhyay, and Ashworth (2006) have demonstrated that when data is lacking, mood serves as a clue, and this conclusion is consistent with customers’ tendency to grasp actual opinions. These findings show that when additional evidence is absent, people rely on their mood for assessment. According to previous research, evaluating adverse services is difficult for clients ( Morgan & Rao, 2006). As a result, it is hypothesized that mood and perceived service quality in healthcare services have a substantial link.

*H2*: *Mood is positively connected to perceived service quality in adverse services.*

Customer’s emotion is a vital factor in evaluating services that they received ( Tsaur, Luoh, & Syue, 2015). Patients depend on their moods when evaluating healthcare services. Researchers believe that happiness affects PSQ through mood since happy people are more likely to be in a positive frame of mind, and hence are more likely to rate service quality positively ( Hellén & Sääksjärvi, 2011; White, 2006), as they are bound to encounter positive emotional states while in adverse circumstances, a happy customer may be more likely to experience enhanced SQ ( Hellén & Sääksjärvi, 2011). As a result, it is hypothesized that happiness and service quality perceptions in healthcare services are mediated by mood.

*H3*: *The association between happiness and perceived service quality is mediated by mood.*

The impact of healthcare quality on PS has been thoroughly researched by scientists ( Marzo *et al.*, 2021; Akbulut, 2016; Badri, Attia, & Ustadi, 2009; Zineldin, 2006; Amin, & Nasharuddin, 2013). Various researchers in the field of marketing have found hypothetical as well as practical links between SQ and other user behaviors such as satisfaction, value, purchase/revisit intentions, and so on ( Akbolat *et al.*, 2021; Lee & Carter, 2011; Theodorakis & Alexandris, 2008; Kouthouris, & Alexandris, 2005).

The disparity between clients’ thoughts and expectations of the services is referred to as hospital service quality ( Aagja & Garg, 2010). Patients are the hospital’s most valuable asset in a healthcare setting. Hospital service quality has grown increasingly important as a means of satisfying and sustaining patients ( Alhashem, Alquraini, & Chowdhury, 2011; Arasli, Haktan, & Turan, 2008). Scholars have discovered a link between PSQ and PS, demonstrating that if healthcare SQ is higher, patient satisfaction will be higher ( Yesilada & Direktör, 2010; Fatima, Malik, & Shabbir, 2018). PS is utilized to define SQ in a healthcare context. SQ and satisfaction are found to have a substantial relationship ( Shabbir *et al.*, 2016). Furthermore, it is assumed that greater services are required to satisfy customers ( Fatima *et al.*, 2018; Marzo *et al.*, 2021). As a result, a considerable relationship between PSQ and PS in healthcare services is envisaged.

*H4*: *Perceived service quality is positively related to patient satisfaction.*

## Methods

### Study design

This research work is highly focused on patient satisfaction in a healthcare setting to empirically validate the hypotheses framed. A cross-sectional research design was applied in the study.

### Setting

The top four districts of Karnataka, India (Bangalore Urban, Dakshina Kannada, Udupi, and Mysore) were identified based on the Human Development Index (HDI) details made available by the Government of Karnataka ( HDI report, 2015). High HDI was considered a significant inclusion criterion as it strongly captures the three dimensions of health, literacy, and standard of living. Research evidence suggests a strong association between HDI and happiness and a stronger positive relationship between HDI and life satisfaction. There is a significant positive association between HDI and the happiness index of a region as reported by Leigh and Wolfers (2006); UNDP Human Development Report authored by Hall, Helliwell, and Helliwell (2014). Applying the simple random sampling technique, Udupi district was selected for this study. The study was conducted in a tertiary care hospital located in this district from November to December 2021.

### Participants

The participants included in the study were outpatients who had more than two visits to the hospital, were aged between 18-65 years, and spoke English or Kannada. The outpatient departments (OPDs) considered for the study were medicine and medical specialties and surgery and surgical specialties. Pediatric and psychiatric OPDs were excluded. Participants were approached at the pharmacy, which is their final point of outpatient service encounter at the hospital.

The sample size was calculated based on the number of items on the rating scale which is multiplied by 10 ( Hair, Sarstedt, Ringle, & Gudergan, 2017) i.e. 17*10 = 170. Accounting for a non-response rate of 20%, 170+34=204. So, it was approximated to 210. The total sample size of the study was 227.

### Ethics and consent

Ethical approval was obtained from the Institutional Ethics Committee (IEC) of Kasturba Medical College and Kasturba Hospital Manipal, Karnataka, India (IEC: 868/2020). Total confidentiality of the data is maintained by not using participant identifierss. This has been included in the participant information sheet (Clause No.12) **.**

### Data collection

Primary data was collected through a structured questionnaire. The questionnaire contained a set of scales evaluating the level of happiness of the participants ( Lyubomirsky & Lepper, 1999). This was rated on a seven-point Likert scale. The set of scales evaluating participants’ mood ( Peterson & Sauber, 1983), service quality perceptions ( Brady & Cronin, 2001; Parasuraman, Zeithaml, & Berry, 1988), and patient satisfaction ( Greenfield & Attkisson, 1989; Oliver, 1997) were rated on a five-point Likert scale. The questionnaire had a total of 23 questions of which 6 captured demographic details of the participants, and 17 were related to the constructs of the study. After the questionnaire was finalized, it was translated into Kannada by a language expert. Both the English and Kannada versions of the questionnaire were created using Microsoft Word 2013, then printed for participant use. This hardcopy is used to collect data from the participants. Data collection was carried out at the hospital pharmacy since it is the last point of contact in outpatient services. The participants were selected purposively. They were informed about the purpose and procedures of the study through a participant information sheet. Written consent was obtained from the participants and then the researcher distributed the questionnaire to participants. The researcher instructed the participants to tick the appropriate response on the questionnaire.

### Pilot testing

Before collecting the data, a pretest procedure was carried out which involved pre-testing of a survey questionnaire to evaluate the complete questionnaire using validity and reliability checks. Validity is the accuracy with which an instrument measures what it is supposed to measure. In this research endeavor, the following validity checks have been implemented.

Face validity: This was checked to see whether at face value the questions/items appeared to be measuring the construct or what is intended to measure.

Content validity: A panel of judges who were experts in healthcare management, marketing, and operations evaluated the draft questionnaire. The survey items were rated based on the clarity, relevance, appropriateness, and redundancy of the items. Out of the 10 experts approached, six of them responded with their comments and suggestions. Suggestions given were incorporated.

Construct validity: This involved convergent and divergent validity checked after receiving data from the final sample.

The data was collected from 47 respondents during the pilot study. Data was collected through a structured questionnaire. A copy of the questionnaire can be found in the *Extended data* ( Akthar *et al*., 2022). The questionnaire was administered personally. The written consent was obtained from the participants before giving the questionnaire. The respondents were approached in September 2021.

Reliability is the consistency or repeatability of the measure. The pilot study helped to determine the construct reliability.

Internal consistency: The reliability within a scale was checked to see whether all the items were designed to measure a particular construct. The reliability scores, Cronbach’s α value of the constructs are as follows: Happiness (0.812), Mood (0.643), Perceived service quality (0.756), and patient satisfaction (0.862) and all the values are well above the threshold limit ( Hair *et al*., 2017).

While conducting the pilot study it was observed that a few participants perceived difficulty in responding to two questions (Items H3: Some people are generally very happy. They enjoy life regardless of what is going on, getting the most out of everything. To what extent does this characterization describe you? and H4: Some people are generally not very happy. Although they are not depressed, they never seem as happy as they might be. To what extent does this characterization describe you?) pertaining to the construct happiness.

These items were reworded and the cognitive interviewing technique (involving two methods i.e. think-aloud interviewing and probing) was adopted ( Willis, 2004) to check the validity of the statements. In order to check the accuracy of the reworded statements, it was subjected to 10 respondents. The paraphrased statements are as follows- H3: People are mostly happy and enjoy their life no matter what is going on to make the most out of everything. Does this describe you?; H4: People are mostly not very happy and not once appear happy as they may be. Does this describe you?

### Analysis

Descriptive statistics were calculated using IBM SPSS statistics 27 (IBM SPSS Statistics, RRID: SCR_016479; Armonk, NY: IBM Corp). The proposed hypotheses were tested and the mediation analysis was performed using the SmartPLS 3 (SmartPLS, RRID: SCR_022040) **.** The results are represented in the form of tables and figures in the subsequent section. SmartPLS 3.0 software was used to analyze the data of this research endeavor. “Partial Least Squares Regression-Structural Equation Modeling” (PLS-SEM) adopts the SEM technique and has many similarities to regression. In addition, PLS also models the theoretical association between the latent variables and also the relationship between the latent variable and its indicators ( Chin, Marcolin, & Newsted, 1996). PLS was also preferred to other covariance-based techniques, like LISREL, as it can be run on smaller sample sizes.

## Results

Descriptive statistics are estimated and the output is presented in Tables 1 & 2. The full dataset can be found in the *Underlying data* ( Akthar *et al*., 2022).

**Table 1.** Demographic characteristics (N=227).

Characteristics	Components	N	%
Gender	Male	97	42.7
	Female	130	57.3
Marital status	Single	51	22.5
	Married	175	77.1
	Divorced/Widowed	1	.4
Age	18-25	35	15.4
	26-40	109	48.0
	41-55	67	29.5
	56-65	16	7.0
Education level	Up to 12 ^th^	86	37.9
	Graduate	113	49.8
	Postgraduate	28	12.3
Occupation	Unemployed	85	37.4
	Employed	73	32.2
	Professional	43	18.9
	Business	26	11.5
Monthly income	25000 and below	108	47.6
	25001-75000	91	40.1
	75001-125000	20	8.8
	125001-200000	8	3.5
	Above 200000	0	0

**Table 2.** Outpatient departments (OPDs).

Category	OPDs	N	%
Medicine	Medicine	47	20.7
Medical Specialties	Dental	19	8.4
	Dermatology	18	8.0
	Endocrinology	9	4.0
	ENT	4	1.8
	Eye	2	.9
	Gastroenterology	9	4.0
	Nephrology	14	6.2
	Neurology	16	7.0
	OBG	14	6.2
	Oncology	5	2.2
	Ophthalmology	10	4.4
	Cardiology	9	4.0
	Pulmonology	17	7.5
	Urology	11	4.8
	Ortho	20	8.8
Surgery	Surgery	2	.9
Surgical specialties	Cardiothoracic	1	.4

**Figure 2.**
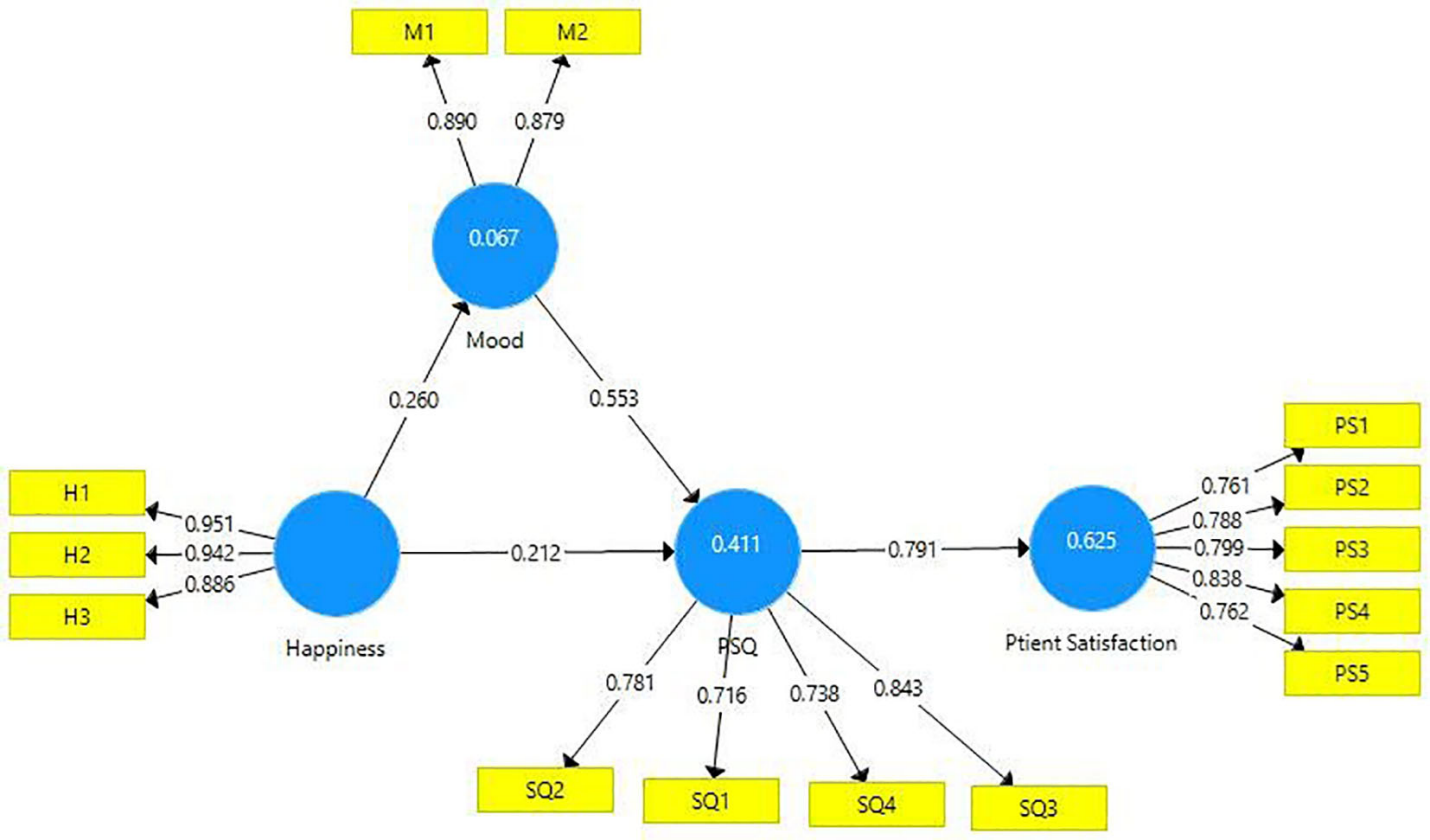
Structural model.

The measurement model was estimated using the data and it is presented below ( Figure 2).

The construct reliability was established by estimating Cronbach’s alpha, factor loadings, and composite reliability ( Table 3). Composite reliability is said to be a better accurate measure of internal consistency as the measure of composite reliability doesn’t tend to increase with the addition of every new item. The threshold value of internal consistency reliability is 0.8 ( Daskalakis & Mantas, 2008), which is established in this research endeavor. In a reflective model, the outer loadings of all indicators have to be above 0.7 ( Henseler *et al.*, 2014), which is evident in this case. Further, the “average variance extracted (AVE)” of each construct must be above 0.5 ( Wasko & Faraj, 2005; Wixom & Watson, 2001) indicating at least 50 percent variance of each construct could be explained by the indicator. These criteria have been fulfilled and presented in Table 3.

**Table 3.** Measurement model evaluation.

Construct	Indicators	Outer loading	Composite reliability	AVE	Cronbach’s alpha	Outer weight	VIF
Happiness	H1	0.951 ***	0.948	0.859	0.918	0.357 ***	3.886
	H2	0.942 ***				0.401 ***	3.119
	H3	0.886 ***				0.319 ***	2.569
Mood	M1	0.890 ***	0.978	0.782	0.721	0.578 ***	1.461
	M2	0.879 ***				0.553 ***	1.467
Patient satisfaction	PS1	0.761 ***	0.854	0.594	0.771	0.249 ***	1.699
	PS2	0.788 ***				0.272 ***	1.839
	PS3	0.799 ***				0.258 ***	1.943
	PS4	0.838 ***				0.247 ***	2.303
	PS5	0.762 ***				0.240 ***	1.726
Perceived service quality	SQ1	0.716 ***	0.892	0.624	0.849	0.278 ***	1.420
	SQ2	0.781 ***				0.347 ***	1.506
	SQ3	0.843 ***				0.356 ***	1.842
	SQ4	0.738 ***				0.311 ***	1.476

*** p<0.01.

** p<0.05.

* p<0.1.

“Discriminant validity” is verified by comparing the AVEs with the squared multiple correlations of each latent variable ( Chin, 1998). In this analysis, Fornell and Larcker criterion is adopted ( Table 4).

**Table 4.** Squared multiple correlations (SMC).

	Happiness	Mood	Perceived service quality	Patient satisfaction
Happiness	0.927			
Mood	0.260	0.884		
Perceived service quality	0.355	0.608	0.791	
Patient satisfaction	0.297	0.593	0.771	0.790

As the AVE of each construct is higher than the squared multiple correlations, it is concluded that the constructs of this research endeavor exhibit discriminant validity. Collinearity among the constructs was tested using the “Variance Inflated Factor (VIF)” guidelines. The predictor variables displayed VIF values below 5 ( Table 3). This implied that collinearity is not a constraint in this structural model.

Hypotheses proposed in this study were examined by comparing the direct effect of patients’ happiness level on PSQ and the indirect effect of the level of patients’ happiness on PS. The results are displayed in Table 5. H1 proposed that the patient’s happiness level positively influences mood and it is supported (β=0.26, t=3.770, p<0.01). H2 proposed that mood positively influenced the PSQ and is supported (β=0.552, t=10.957, p<0.01). H3 proposed that happiness positively influenced PSQ and this hypothesis is also supported (β=0.212, t=3.958, p≤0.01). H4 proposed a direct positive effect of PSQ on PS (β=0.791, t=29.516, p≤0.01). The path values (β values) and the empirical t values of all the hypotheses are above the cutoff value of 0.2 and 1.96 respectively, which substantiates the proposed hypotheses of this research endeavor. The influence of all the exogenous latent variables namely, happiness, mood, perceived service quality, on the endogenous latent variable of patient satisfaction is estimated to be moderate (R ^2^=62.5%) ( Hair *et al*., 2017).

**Table 5.** Hypothesis testing, f ^2^.

Relationship	Path coefficient	t-Value	Bias Corrected 95% Confidence Interval	f ^2^
Happiness - mood	0.260 ***	3.3770	(0.111,0.409)	0.072
Happiness - perceived service quality	0.212 ***	3.958	(0.106, 0.313)	0.071
Mood - perceived service quality	0.553 ***	10.957	(0.736, 0.651)	0.484
Perceived service quality - patient satisfaction	0.791 ***	29.516	(0.736, 0.846)	1.669

*** p<0.01.

** p<0.05.

* p<0.1.

The effect size, f ^2^ of all the exogenous latent variables was calculated ( Table 5). The effect size measures the extent of influence of the variables independent of the scope of the sample analyzed ( Cohen, 1988). Cohen (1988) proposes a threshold to gauge the extent of the effect of the constructs. Effect size above 0.35 is reported as a large effect; value in the range of 0.15 to 0.35 is reported as moderate effect and values below 0.15 is reported as a low effect. In our research endeavor, the effect size of mood on PSQ (f ^2^=0.484) and the effect size of PSQ on PS (f ^2^=1.669) are estimated to be large. Model Fitness is assessed with the help of the value of “Standard Root Mean square Residual (SRMR)” as proposed by Henseler *et al.* (2014). The threshold value of model fitness is 0.8 ( Hu & Bentler, 1998). The SRMR value of this model is reported as 0.073 which indicates a good model fit.

**Figure 3.**
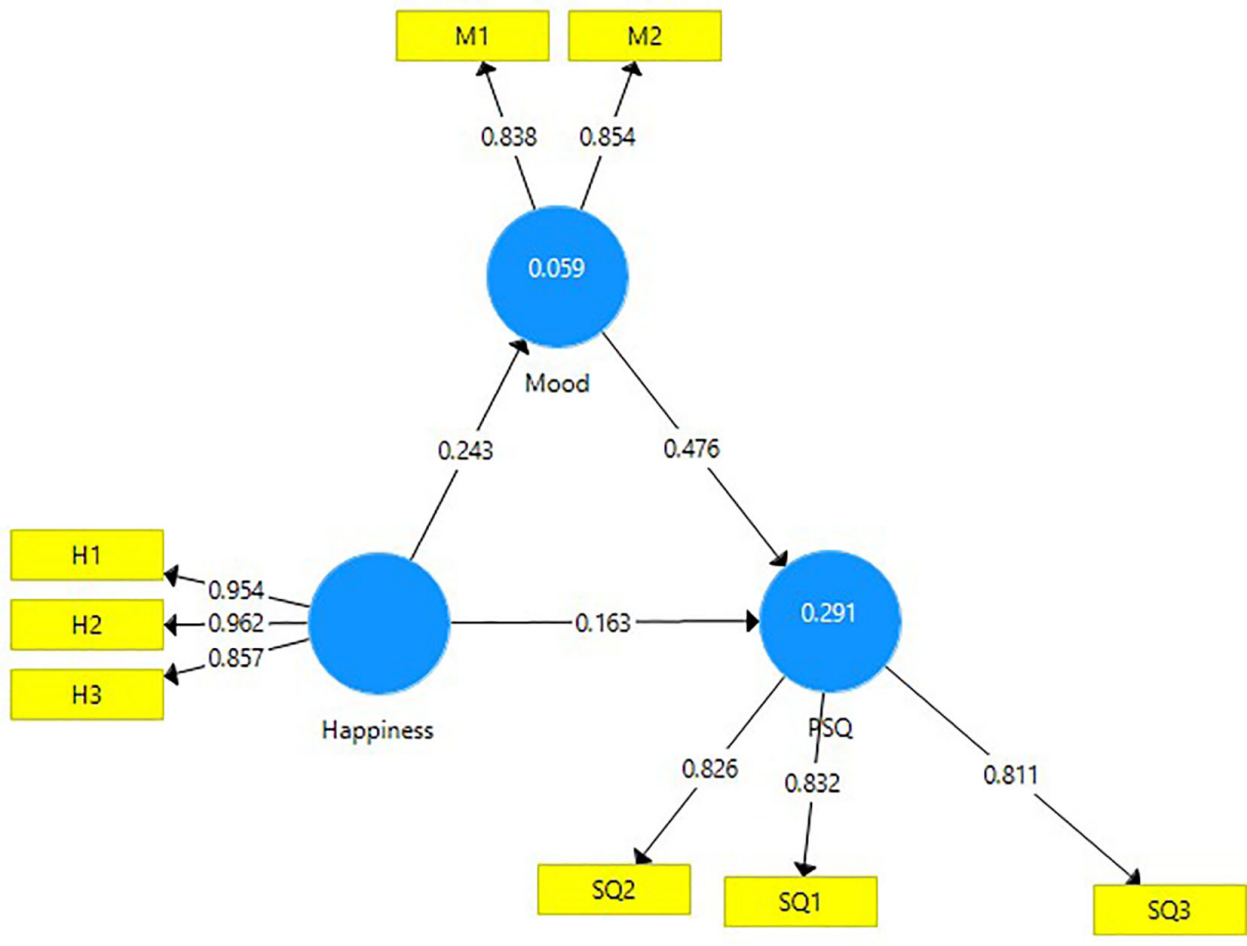
Mediation analysis.

A mediation analysis was undertaken to assess the mediating effect of the construct ‘mood’ between the constructs of happiness and PSQ ( Figure 3).

The direct effect of happiness on perceived service quality (0.163) is significant and the indirect effect of happiness on PSQ through mood (0.116) is also significant. The VAF (Variance Accounted for) value of 41.58 percent indicates a partial mediation between happiness and PSQ ( Table 6).

**Table 6.** Mediating effect of Mood.

	Direct effect	Indirect effect	Total effect	VAF	Mediation
Figure 3	0.163 ***	0.116 ***	0.882 ***	41.58%	Partial

*** p<0.01.

** p<0.05.

* p<0.1.

### Importance performance matrix analysis (IPMA)

Importance Performance Matrix Analysis (IPMA) provides researchers with an insight into the relative importance of the performance of the exogenous latent variables in their association with the endogenous latent variables and was first proposed by Martilla and James (1977). This method enables the researchers to examine the importance of an item in addition to its performance. The rationale of this analysis is to identify the total effect of the predecessor constructs (mood, perceived service quality, and happiness) in forestalling the target endogenous construct (patient satisfaction) ( Hair, Hult, Ringle, & Sarstedt, 2016, p. 276; Hair, Sarstedt, & Ringle, 2018, p. 105). The total effect establishes the importance of the constructs while the mean value of their scores reflects their performance (ranging from 0, which is the lowest, to 100, which is the highest) ( Höck, Ringle, & Sarstedt, 2010, p. 201).

**Figure 4.**
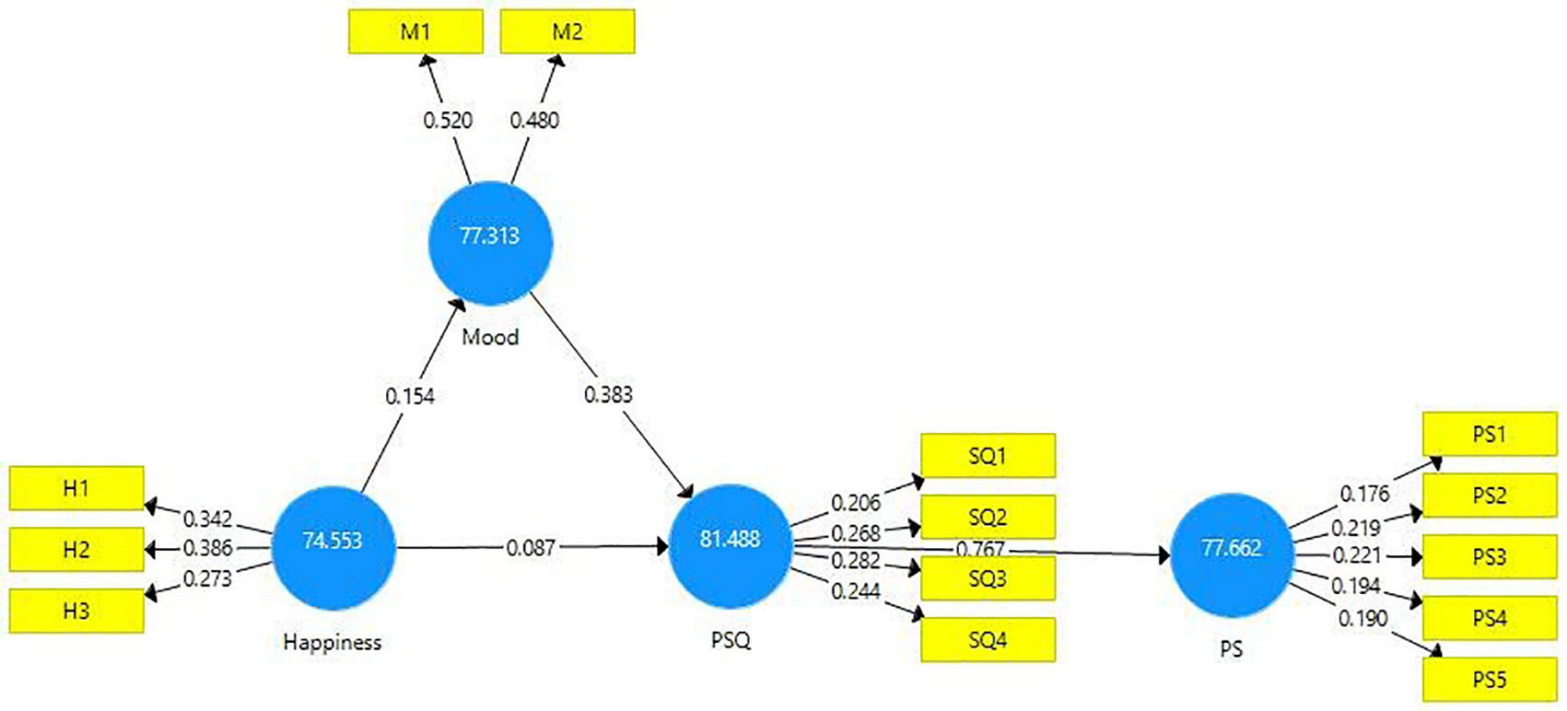
Importance performance matrix analysis.

The results of IPMA are presented in Figure 4 and Table 7. Analyses demonstrate that PSQ is ranked high on performance (81.48) in comparison to the other exogenous constructs. In addition, the total effect of PSQ on PS is 0.791 which is also high. Thus, a unit increment in the performance of PSQ from 81.448 to 82.448 will result in an increase in the performance of PS from 77.662 to 78.453. The total effect and performance of the exogenous construct mood are 0.437 and 77.313 respectively. Thus one unit increment of mood from 77.313 to 78.313 would increase the performance of PS from 77.662 to 78.099. Similarly, the total effect and performance of the exogenous construct happiness are 0.281 and 74.553. Thus, an increment of one unit of happiness from 74.553 to 75.553 would yield an increment in patient satisfaction to 77.943. This study found that the total effect of PSQ has the strongest and most significant effect on patient satisfaction followed by Mood and then Happiness. This is an important implication to healthcare service providers.

**Table 7.** Importance performance matrix analysis of institutional effectiveness.

Latent constructs	Patient satisfaction	
	Importance (total effects)	Performance (index values)
Happiness	0.281	74.553
Mood	0.437	77.313
Perceived service quality (PSQ)	0.791	81.488

**Figure 5.**
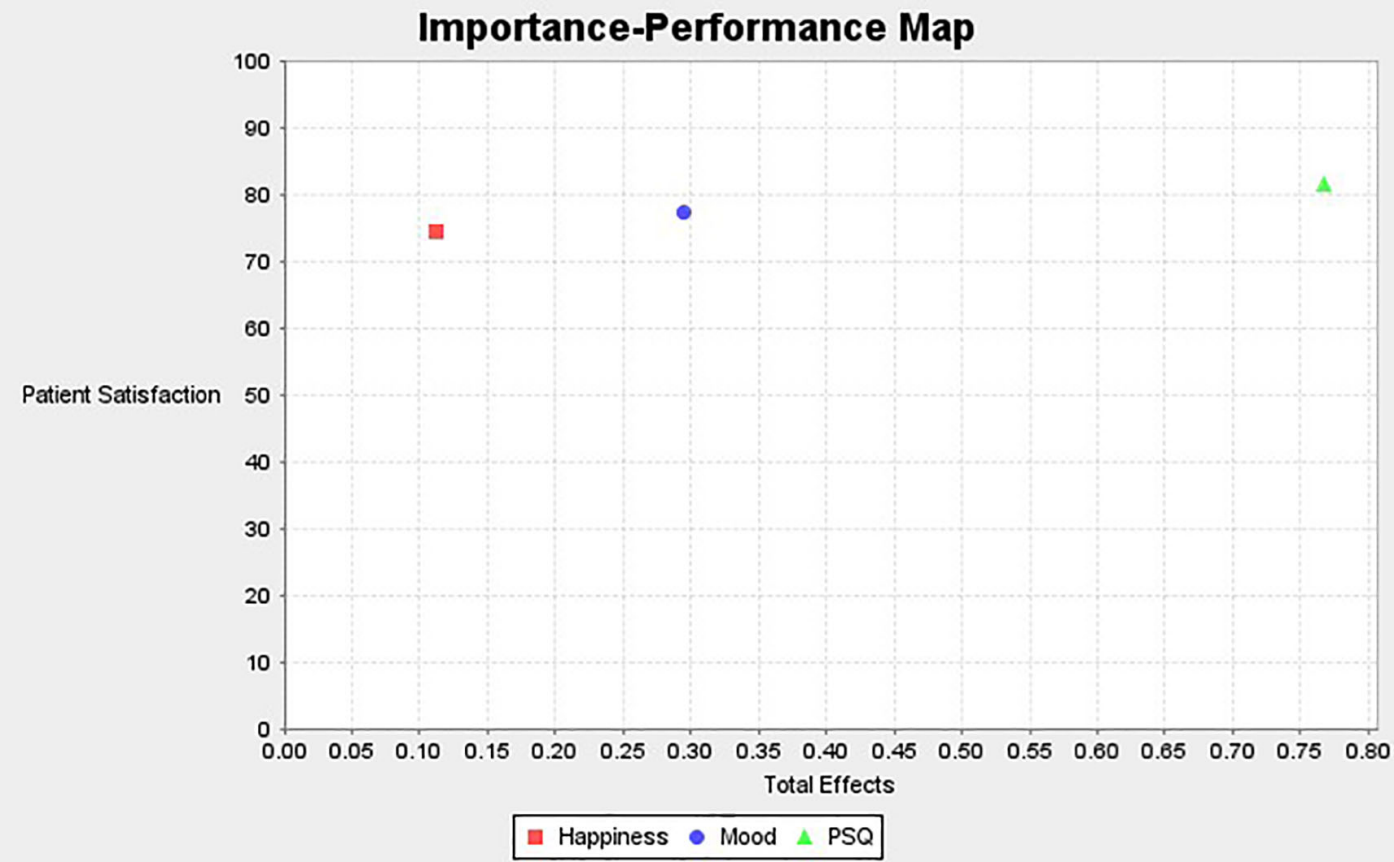
Importance performance map.

The Importance Performance Map is presented below ( Figure 5).

## Conclusions/Discussion

In this research endeavor, we intended to explore the role of emotions in adverse services. We proposed to explore if mood mediates the relationship between happiness and service quality perception which had a direct bearing on patient satisfaction. Happiness was explored as a significant antecedent to perceived service quality of adverse services, such as hospitals, and it provides a significant foundation to determine patient satisfaction. These findings enable us to conclude that happy people are most likely to experience better service quality and patient satisfaction. We can also conclude that patients’ emotions (mood) at the hospital play an instrumental role in developing service quality perceptions and indirectly strengthening patient satisfaction.

The results of this research endeavor to uphold the results of previous research from positive psychology and service marketing literature showing that the happiness of people significantly influences their mood which directly or indirectly influences their service quality perception, especially in adverse services such as hospitals, legal services, etc ( Morgan & Rao, 2006; Diener *et al.*, 2009; Hellén & Sääksjärvi, 2011; Badri *et al.*, 2009; Yesilada & Direktör, 2010; Fatima *et al*., 2018). However, this research endeavor extends the literature by displaying that happiness is a significant predictor of mood and mood mediates the association between happiness and perceived service quality in adverse services. This study contributes to the body of knowledge by highlighting the role of patients’ moods in predicting service quality and thereby patient satisfaction.

These results could be used to deduce the following. Firstly, happy people would experience positive affective states, and consequently, a person who scores high on happiness is more likely to report a positive mood. Secondly, the mood is a reflection of their happiness and hence the results could be biased ( Diener, 2009). This study contributes to the body of knowledge by highlighting the role of patients’ moods in predicting service quality and thereby patient satisfaction. This result does not echo the outcomes of past research such as Kumar and Oliver (1997) and Oliver (1993), who proposed that there is no significant relationship between consumers’ affective response and service quality. However, White (2006) has proposed a positive association between mood and service quality, and our findings are in line with this research output.

This research also presents significant managerial implications to industry, especially to the services of adverse nature. We recommend that marketers of adverse service must design strategies to enhance the mood of their patients or customers. Every element of the servicescape in adverse services must be designed such that it enhances the mood of customers. From service providers (doctors and nurses) and support staff to peripheral service encounters, there should be effective management that contributes to elevating the customer’s mood. Doctors and nurses can be trained to handle customers’ queries about the line of treatment and medication effectively. Health care providers or medical teams, environmental conditions, and hospital completeness are all elements that influence patient satisfaction. To enhance patient satisfaction, the quality of care provided by health services, human resources, and infrastructure must be improved. As a result, the entire service encounter can be made more enjoyable for the customers by reducing the distress caused by adverse services.

This study is also subject to limitations. First, this study was conducted in a tertiary hospital of a high HDI district. There is a significant positive association between HDI and the happiness index of a region as reported by Leigh and Wolfers (2006). Future research would benefit from conducting a comparative study amongst high HDI and low HDI districts. Second, this study adopted a quantitative approach. Future researchers would consider improving patient satisfaction by exploring the elements of servicescape in adverse services through an experimental approach. The study design adopted in positive psychology research endeavors consists of a two-step process. In the preliminary stage, happy and unhappy subjects are identified and are then subject to the experimental setting. This paves the way for effective comparison in both groups. In this scenario, service quality perceptions could have been effectively captured in the controlled group and the experimental group. Thirdly, this study utilized the scale developed by Peterson and Sauber (1983) to measure the construct mood which might be a cause of concern. This scale fails to capture the extent of influence of elements of servicescape on a patient’s general mood. Thus there could be a possibility of an element of error in capturing the patient’s mood. However, it can also be argued that a patient’s mood captured at the hospital is attributed to elements of servicescape.

In conclusion, the concept of consumer emotions and its implication on service quality evaluation had gained momentum. Taking a step in this direction, this research endeavor explored the impact of consumers’ happiness on service quality perception at a hospital, which is considered to be an adverse service by nature. This research outcome indicated that consumers’ mood partially mediated the association between happiness and service quality perception. This outcome provides significant evidence that goes against the theoretical underpinnings of positive psychology theories which suggest that happy people significantly experience situations more positively. However, outcomes of this research endeavor contribute to service marketing literature which highlights the role of servicescape to modify the mood of consumers thereby influencing their service quality perception. Hospitals must design their servicescape effectively, to trigger positive emotions (mood) among patients that will have a direct bearing on service quality evaluation and thereby patient satisfaction. This is of paramount importance, especially during the current COVID-19 pandemic. COVID-19 has created significant distress economically and emotionally across the globe. Mental health has become the focal point of discussion and concern. Hence, hospitals must ensure that all their marketing strategies revolve around creating a positive affective state (mood) among their patients which will enable them to perceive adverse services in hospitals in a positive way.

## Data availability

### Underlying data

Figshare: Underlying data: Can positive emotions predict consumer satisfaction in adverse services? An empirical investigation. https://doi.org/10.6084/m9.figshare.19360625.v3 ( Akthar *et al*., 2022)

This project contains the following underlying data:

- Dataset.csv

### Extended data

This project contains the following extended data:

- Questionnaire.docx
- Informed Consent.docx
- Participant Information Sheet.docx

Data are available under the terms of the Creative Commons Zero “No rights reserved” data waiver (CC0 1.0 Public domain dedication).
