# Supplementary material for: Can Positive Emotions Predict Consumer Satisfaction in Adverse Services?
Source: F1000Res. 2024 Jan 12;11:347. Originally published 2022 Mar 22. [Version 2] doi: 10.12688/f1000research.110256.2 (PMC10776967; doi:10.12688/f1000research.110256.2)
Supplement: Peer review history

# Reviewer report: Reviewer response for version 1

**DOI:** 10.5256/f1000research.121839.r162772

**Published:** 2024-01-09

**Authors:** Blessy Prabha Valsaraj

**Affiliations:** 1 Department of Community and Mental Health, College of Nursing, Sultan Qaboos University, Seeb, Oman

The article is well written. Methodology can be more explained, the design, setting (about the hospital), data collection process etc.

 Suggestions: 

 Can add more recent literature.

 UNDP Human Development Report :

 Setting: Expand UNDP at the first usage in the article.

 Possible limitations of the results:

 1. Happiness is one type of mood itself, so I think these two variables are greatly overlapped, further proven by the positive correlation in the study.

 2. Mood can be a reflection of the person's personality trait, such as perceiving the world around as positive and thus assuming optimistic perspectives on all the circumstances in life, including adverse. But is known that  environment also has an impact in the mood and happiness of the individual. Thus, it could be due to the positive hospital environment (being one of the HDI) it would have triggered the happiness? (A random selection of the hospitals and the settings would have minimized this bias).

Is the work clearly and accurately presented and does it cite the current literature?

Partly

If applicable, is the statistical analysis and its interpretation appropriate?

Yes

Are all the source data underlying the results available to ensure full reproducibility?

Yes

Is the study design appropriate and is the work technically sound?

Yes

Are the conclusions drawn adequately supported by the results?

Yes

Are sufficient details of methods and analysis provided to allow replication by others?

Yes

Reviewer Expertise:

Mental Health

I confirm that I have read this submission and believe that I have an appropriate level of expertise to confirm that it is of an acceptable scientific standard.

# Response

**Published:** 2024-01-10

**Authors:** Nahima Akthar

**Affiliations:** Manipal Academy of Higher Education, India

Dear Reviewer, We appreciate the time taken by you to go through our article and provide a detailed review of the article. We have incorporated all the changes suggested by you. The response to the reviewer's comments is mentioned below:

 1. Thank you for your suggestion. The methodology section has been revised as suggested.

 2. This article was submitted in February 2022. Therefore, the articles published till 2021 which are related to this study were included.

 3. Thank you for the suggestion. This has been incorporated as suggested.

 4. Thank you. When we look at the variables happiness and mood, they look similar. However, as per positive psychology literature happiness and mood are two different constructs. Hence, we have proved statistically that there is a significant relationship between happiness and mood. 

 5. Thanks for the suggestion. Yes. A positive hospital environment does impact patient's happiness and mood. In this research, the hospital is selected through a simple random sampling.

# Reviewer report: Reviewer response for version 1

**DOI:** 10.5256/f1000research.121839.r130100

**Published:** 2022-04-22

**Authors:** Doraiswamy Ashok

**Affiliations:** 1 VIT Business School, Vellore Institute of Technology, Vellore, India

I am very much pleased to add my review report on the title 'Can positive Emotion predict consumer satisfaction in adverse services' submitted by Nahima Akhar, Dr. Sunitha  Naayak and Yogesh Pai P. 

 First of all, I congratulate the authors for choosing the topic, which is more beneficial to the society at large. The sequence of the paper is well prepared. The reviews conducted for the research gap are also self demonstrative, the methodology and tools used in this study add more relevance to the present context. 

 The analysis part of the paper sounds good. The interpretations given about the study results project the validity of the paper to all stakeholders involved in it.  

 The entire article was prepared without any flaws in the language used and was also informative, to the entire researchers' community. 

 The themes of the paper showed unique by comparing the direct effect of patients' happiness level on perceived service quality and the indirect effect of the level of patients' happiness on patients' satisfaction. Further, this study also compared all the exogenous latent variables like happiness, mood, and perceived service quality with the endogenous latent variables of patient satisfaction.  

 At the outset, the study conducted demonstrated how patients satisfaction is dependent on patients' happiness. The implication of the study is also well explained. 

 Once again by congratulating the authors of the study, I place my recommendation for indexing the article.

Is the work clearly and accurately presented and does it cite the current literature?

Yes

If applicable, is the statistical analysis and its interpretation appropriate?

I cannot comment. A qualified statistician is required.

Are all the source data underlying the results available to ensure full reproducibility?

Yes

Is the study design appropriate and is the work technically sound?

Yes

Are the conclusions drawn adequately supported by the results?

Yes

Are sufficient details of methods and analysis provided to allow replication by others?

Yes

Reviewer Expertise:

I am basically environmental science at the postgraduate level with a doctorate in Management studies. I am presently working as a professor, in management studies at VIT Business School, VIT, Vellore.

I confirm that I have read this submission and believe that I have an appropriate level of expertise to confirm that it is of an acceptable scientific standard.

# Response

**Published:** 2022-04-23

**Authors:** Nahima Akthar

**Affiliations:** Manipal Academy of Higher Education, India

Dear reviewer, we appreciate the time taken by you to go through our article and submit a detailed review of it. We will go through the paper again and further check for any lapses, and rectify them if any. Thank you.
